# Radiomics analysis of biplanar ultrasound images can discriminate non-mass breast carcinoma from mastitis

**DOI:** 10.3389/fonc.2026.1785714

**Published:** 2026-07-01

**Authors:** Qinfu Wu, Guangde Liu, Mengqiang Xiao, Shanghuang Xie, Wenhui Teng, Yang Dong, Xiaoyi Chen, Tianzhu Liu, Peikai Huang

**Affiliations:** 1 Department of Ultrasound, Zhuhai Hospital, Guangdong Provincial Hospital of Traditional Chinese Medicine, Zhuhai, China; 2 Department of Radiology, Guangdong Hospital of Traditional Chinese Medicine, Zhuhai, China; 3 Lab of Molecular Imaging and Medical Intelligence, Department of Radiology, Longgang Central Hospital of Shenzhen/Shenzhen Clinical Medical College, Guangzhou University of Chinese Medicine/Longgang Clinical Institute of Shantou University Medical College, Shenzhen, China

**Keywords:** clinical–radiomics strategy, mastitis, non-mass breast carcinoma, radiomics, ultrasound

## Abstract

**Background:**

Non-mass breast carcinoma (NMBC) is a diagnostically challenging subtype with sonographic features that often overlap with mastitis, leading to misdiagnosis and delayed treatment. Unlike mastitis, which typically warrants conservative therapy, NMBC requires prompt oncological management, making accurate preoperative differentiation critically important. Using transverse, longitudinal, and fused biplanar ultrasound images, we constructed radiomics models to distinguish NMBC from mastitis and evaluated the incremental diagnostic value of combining radiomics features with clinical variables.

**Methods:**

We retrospectively analyzed data from 139 patients (NMBC, 63; mastitis, 76) and extracted radiomics features from manually segmented transverse and longitudinal ultrasound images. After feature stability screening and least absolute shrinkage and selection operator selection, we constructed three radiomics logistic regression models for transverse, longitudinal, and fused imaging data, respectively, and additionally developed clinical variable-based models (age and BI-RADS) and combined clinical–radiomics models. The models were trained and internally validated using a 70/30 split, performance was assessed based on area under the curve (AUC), accuracy, sensitivity, specificity, and decision curve analysis (DCA) values.

**Results:**

In the training cohort, the fusion model (combining features from transverse and longitudinal images) outperformed single-plane models (AUC = 94.2%, accuracy = 87.6%). However, this advantage diminished in the validation cohort, where the transverse, longitudinal, and fusion radiomics models showed comparable performance (AUC = 0.730, 0.823, and 0.800, respectively; accuracy = 69.0%, 78.6%, and 78.6%, respectively). DeLong test revealed no significant differences among the models in the validation cohort. The combined clinical–radiomics model outperformed both radiomics and clinical variable-based models (AUC: 0.861–0.884 in the validation cohort); likelihood ratio tests confirmed that radiomics features and clinical variables contributed independently to the prediction. Calibration and DCA revealed that the fusion model showed superior clinical utility in the training cohort but demonstrated limited generalizability in the validation cohort.

**Conclusion:**

Biplanar ultrasound imaging-based radiomics models show promise for distinguishing NMBC from mastitis. Although the fusion model excelled in the training cohort, it offered limited advantage internal hold-out validation. Integrating clinical variables with radiomics features offers added diagnostic value beyond either approach alone, suggesting that a combined clinical–radiomics strategy may be more clinically viable.

## Introduction

Breast cancer is the leading malignancy among women globally, comprising ~11.7% of incident cancer cases among women (1). Approximately 1.3% of breast malignancies detected *via* ultrasound are non−mass breast carcinomas (NMBCs) (2). Although their identification predominantly relies on imaging, particularly ultrasound, they are often misdiagnosed or underdiagnosed because of the overlap in their ultrasound features with those of mastitis (3). The consequently suboptimal diagnostic accuracy during screening represents a major hurdle in ultrasound practice.

Radiomics is a data-driven imaging tool that obtains high-throughput features from radiological images, revealing visually imperceptible tumor heterogeneities (4). It facilitates linking imaging phenotypes to underlying pathology (5, 6), tumor behavior, and treatment response (7), which gives it strong biological and clinical relevance. Therefore, radiomics has improved diagnostic accuracy and reduced interobserver variability in breast imaging, particularly in ultrasound assessments (8).

Recent research has highlighted its diagnostic potential in breast tumors. Gu et al. emphasized on the role of radiomics in personalized breast management (9). Besides, in the three predictive models developed by Gong et al. (10), the combined model showed an area under the curve (AUC) value of 0.964 in the validation cohort, outperforming both traditional Breast Imaging Reporting and Data System (BI-RADS) assessment and radiomics alone. In a different study, Gong et al. (11) used radiomics to identify malignancies and classify molecular subtypes on the basis of both conventional and contrast-enhanced ultrasound images; they reported AUC values of 0.978 and 0.956 in the training and validation cohorts, respectively, demonstrating high diagnostic accuracy with only a minor decline from training to validation, suggestive of good model generalizability.

However, as most studies have primarily focused on lesions presenting as masses, non-mass lesions like NMBCs remain underexplored. Notably, these lesions typically lack distinct margins and can mimic benign conditions like mastitis; this complicates the diagnostic process. Although the accessibility and non-invasiveness of ultrasound make it a primary imaging tool for breast evaluation, its diagnosis—particularly for non-mass lesions—is highly subjective and operator-dependent (12–15). Moreover, studies exploring the utility of MRI-based radiomics to differentiate between inflammatory breast cancer and mastitis have been limited by small datasets and lack of external validation (16, 17). To address this clinical gap, we used radiomics models based on both transverse and longitudinal ultrasound images to differentiate NMBC from mastitis. We also evaluated their diagnostic performance using machine learning.

## Materials and methods

### Ethics approval

This retrospective study—approved by the Medical Ethics Committee of the Guangdong Provincial Hospital of Chinese Medicine (approval no.: ZE2025-433)—was performed in compliance with the principles of the Declaration of Helsinki. As the analysis included de-identified retrospective data which posed negligible individual risk, the need for written informed consent was waived. Personal identifiers were removed from all datasets, and no additional procedures were performed.

**Figure 1:**
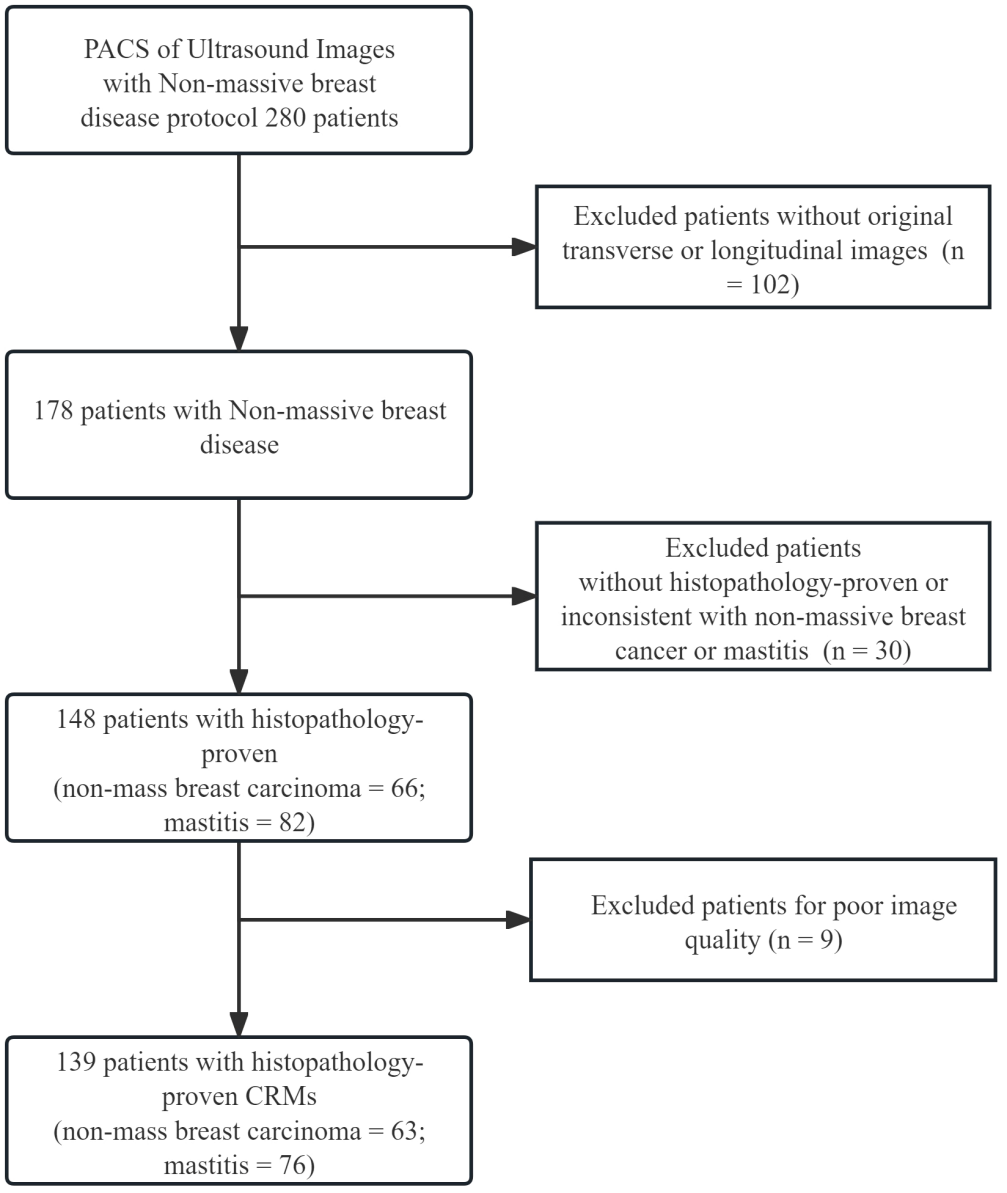
Patient distribution and definition of the training and validation datasets.

Data from 139 patients with either pathologically confirmed NMBC (n = 63) or mastitis (n = 76) were retrospectively analyzed. The patients received treatment between January 2018 and February 2024. We included only cases for which both transverse and longitudinal ultrasound images were accessible from the Picture Archiving and Communication System (PACS) and for which complete clinical data—including lesion location, biopsy or surgical records, and histopathological findings—were available. Patients with suboptimal or insufficient ultrasound data and those with lesions other than NMBC and mastitis were excluded. **Figure 1** shows the patient selection process.

### Ultrasound examination protocol

For breast ultrasound imaging, a high-resolution linear-array transducer (12–15 MHz) was used. Patients were examined in the supine or slightly oblique position, with their ipsilateral arm raised. The entire breast and axilla were evaluated in both transverse and longitudinal planes—including the subcutaneous, mammary, and retromammary regions. Suspicious lesions were evaluated in two orthogonal planes, with the focal zone optimized for margin visibility.

To ensure consistent echogenicity, system settings like gain, depth, and time gain compensation were adjusted. Speckle reduction and compound imaging were used to reduce artifacts, and Doppler or microvascular imaging was used to assess vascularity. During Doppler imaging, minimal probe pressure was applied to prevent compression of low-flow vessels. Panoramic imaging was used to evaluate diffuse or non-mass lesions.

The lesion position was documented relative to the nipple and pectoral fascia, with measurements acquired in two orthogonal dimensions. To ensure consistency and reproducibility, transducer orientation and scanning angles were standardized. The Digital Imaging and Communications in Medicine (DICOM) images were stored in the PACS. After thorough review for artifacts, images of suboptimal quality or those containing measurement data were excluded from analysis. Full bilateral examination required 15–20 min.

### Image segmentation

Two experienced sonologists (T.W.H. and Y.D., with 15 and 17 years of experience, respectively) performed image segmentation using the ITK-SNAP interactive software (http://www.itksnap.org). To prevent over- or under-segmentation, the entire lesion was carefully delineated on both transverse and longitudinal images. Each reader performed segmentation independently, and all the contoured images were reviewed by a senior expert (G.D.L. with over 20 years of experience); discrepancies were resolved *via* consensus.

To ensure a consistent standard, resampling was performed on all images and their respective masks, and a uniform pixel spacing of 1 × 1 mm² was maintained. The PyRadiomics package in Python3.9 (Python Software Foundation, Delaware, USA) (18) was used to extract the radiomics features; 756 features belonging to the following seven categories were extracted from each image plane: shape, first-order statistics, and five textural metric families—Gray-Level Co-Occurrence Matrix (GLCM), Gray-Level Run-Length Matrix (GLRLM), Gray-Level Size-Zone Matrix (GLSZM), Neighborhood Gray-Tone Difference Matrix (NGTDM), and Gray-Level Dependence Matrix (GLDM). To compute additional features from each category, a total of 14 filters, including exponential, gradient, square, and wavelet filters, were applied, collectively generating the total of 756 features per image plane. Each feature was normalized using Z-score standardization.

**Figure 2:**
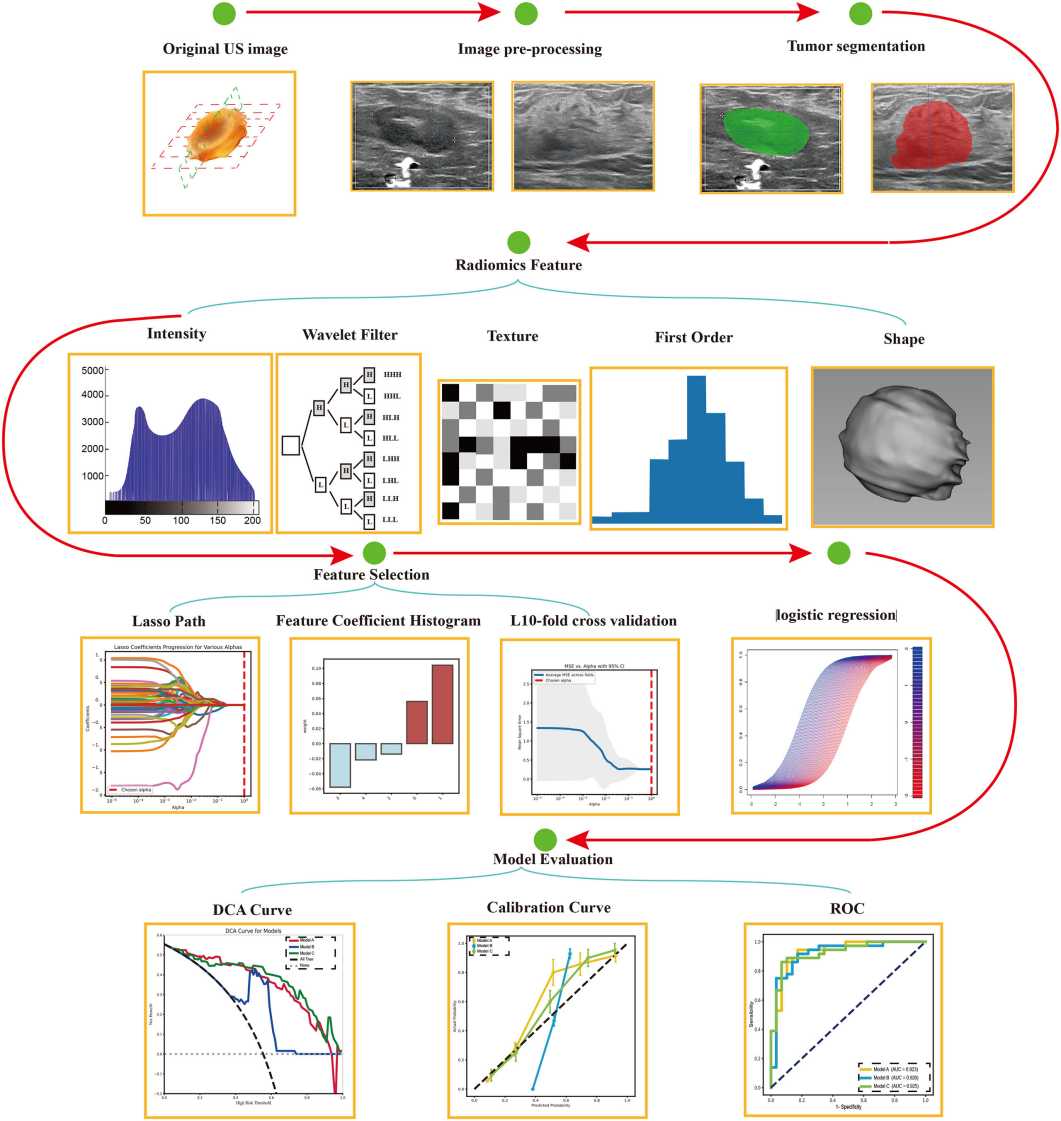
Workflow for the development and evaluation of the logistic regression models.

Potentially significant features were identified using a two-sample t-test. Subsequently, the least absolute shrinkage and selection operator (LASSO) regression with 10-fold cross-validation was applied to refine feature selection and determine optimal regularization parameter. Only features with non-zero regression coefficients were retained for further modeling. The complete radiomics workflow is shown in **Figure 2**.

### Model development

The models were developed using the logistic regression function, and to develop the longitudinal and transverse models, radiomics features from the longitudinal and transverse ultrasound images were used, respectively. For the fusion model, radiomics features from both longitudinal and transverse images were included. Overall, 70% of the cases (43 malignant and 54 benign) were randomly assigned to the training dataset, and the remaining 30% (20 malignant and 22 benign) were assigned to the internal validation set. To evaluate the added value of radiomics features beyond clinical variables, we first constructed three clinical variable-based models (1): an age-only model (2), a BI-RADS-based model using only the binary BI-RADS category (4A/4B/4C = 0; 5/6 = 1) *via* logistic regression, and (3) a clinical model incorporating age and BI-RADS category *via* logistic regression. Then, we developed a combined clinical–radiomics model integrating age, BI-RADS category, and LASSO-selected radiomics features *via* logistic regression.

### Statistical analysis

Statistical analysis was conducted using the SPSS 27.0 (IBM, Armonk, NY, USA) and R 4.2.2 (R Core Team, 2024) software. A two-sided *P* value of <0.05 was considered to indicate statistical significance. To assess differences in demographic and clinical characteristics, the Chi-square or independent-sample t-tests were used, as appropriate. Intra- and inter-reader reliability for image segmentation was evaluated on the basis of the intraclass correlation coefficient (ICC), and only features with ICC values of >0.75 were retained. The discriminative ability of each model was determined based on the accuracy, sensitivity, specificity, area under the receiver operating characteristic curve (AUC), positive predictive value (PPV), and negative predictive value (NPV). Pairwise AUC comparisons were performed using the DeLong test and bootstrap testing with 2,000 resamples. Radiomics feature importance was assessed by deriving the SHapley Additive exPlanations (SHAP) values. Calibration curves were plotted using the rms package, and decision curve analysis (DCA) was performed using the “rmda” and “ggDCA” Python packages. ROC curve analysis was performed using the pROC package. The sensitivity, specificity, accuracy, PPV, NPV, and AUC values were calculated and presented using the reportROC package. To assess whether radiomics features contributed significantly beyond clinical variables, we performed multivariate logistic regression with the radiomics score, age, and BI-RADS as predictors, and we evaluated their significance using likelihood ratio tests.

## Results

### Patient characteristics

Among 139 patients included for this study, 76 (mean age, 34.05 ± 5.23 years) and 63 (mean age, 43.54 ± 10.75 years) patients had mastitis and NMBC, respectively (**Figure 1**). Although significant differences were found in terms of age and BI-RADS scores, no significant differences were noted in lesion location. The most common histologic subtype was IDC-NST (46/63, 73.0%) in the NMBC group and granulomatous lobular mastitis (61/76, 80.3%) in the mastitis group (**Table 1**).

**Table 1:** Clinical and pathological characteristics.

Characteristics	Mastitis	NMBC	*P* value
Age (years), mean ± SD	34.05 ± 5.23	43.54 ± 10.75	<0.05
Age group (years), n (%)			<0.05
<30	16 (21.1%)	8 (12.7%)	
30–39	55 (72.4%)	20 (31.7%)	
40–49	4 (5.3%)	13 (20.6%)	
≥50	1 (1.3%)	22 (34.9%)	
Sex			
Male	0 [0%]	0 [0%]	N/A
Female	76 [100%]	63 [100%]	
Location			
Right breast	42 [55.26%]	32 [50.79%]	0.61
Left breast	34 [44.74%]	31 [49.21%]	
BI-RADS category			
III/IV/V/VI	0/76/0/0	0/48/14/1	<0.05
Histologic subtype	GLM (61)/PCM (3)/Other (12)	IDC, NST (46)/DCIS (8)/IDC+DCIS (4)/ILC (3)/Apocrine DCIS (1)/Other (1)	

BI-RADS, Breast Imaging Reporting and Data System; GLM, granulomatous lobular mastitis; PCM, plasma cell mastitis; IDC, invasive ductal carcinoma; NST, no special type; DCIS, ductal carcinoma *in situ*; ILC, invasive lobular carcinoma.

### Diagnostic performance of radiomics models

In the validation set, the longitudinal, transverse, and fusion radiomics models achieved AUC values of 0.823, 0.730, and 0.800, respectively (**Table 2**). The fusion model showed the highest AUC in the training set (0.942) but a notable decline in the validation set, whereas the longitudinal model exhibited relatively stable performance across both sets. DeLong testing revealed that the fusion model significantly outperformed both single-plane models in the training set (transverse *vs*. fusion: *P* = 0.001; longitudinal *vs*. fusion: *P* = 0.016); however, no significant differences among the three models were observed in the validation cohort (all *P* > 0.05). Detailed performance metrics, including sensitivity, specificity, and accuracy, are provided in **Table 2**. Comparing radiomics models with clinical variable-based models revealed that the age-only model (AUC = 0.717; sensitivity = 60.0%, specificity = 77.3%) outperformed the BI-RADS-only model (AUC = 0.650; sensitivity = 30.0%, specificity = 100.0%), with notably higher sensitivity but lower specificity; the low sensitivity of the BI-RADS-only model was attributable to the fact that BI-RADS ≥ 5 was present in only a subset of NMBC cases. The clinical model (age + BI-RADS) achieved an AUC of 0.715, which was comparable to that of the age-only model (AUC = 0.717), suggesting no incremental value of binary BI-RADS beyond age alone. Notably, across all three feature sets, the combined clinical–radiomics model consistently outperformed both the clinical variable-based and radiomics models, achieving AUC values of 0.873, 0.861, and 0.884 for the transverse, longitudinal, and fusion models, respectively (**Supplementary Figure 2**; **Supplementary Table 2**). Likelihood ratio tests confirmed that the radiomics score remained a significant predictor after adjusting for age and BI-RADS (longitudinal: *P* = 0.010; transverse: *P* = 0.007; fusion: *P* = 0.003), and conversely, clinical variables remained significant after adjusting for radiomics (all *P* < 0.004), indicating complementary diagnostic value. Calibration curves and DCA for the expanded model comparisons are presented in **Supplementary Figure 2**. Bootstrap testing revealed that no pairwise AUC comparisons reached statistical significance (all *P* > 0.05), which is likely attributable to the limited sample size of the validation cohort (n = 42).

**Table 2:** Diagnostic efficiency of the transverse, longitudinal, and fusion ultrasound models in differentiating mastitis (N = 76) from NMBC (N = 63) in the training and validation datasets.

Logistic regression models	Sensitivity (%)	Specificity (%)	Accuracy (%)	PPV (%)	NPV (%)	AUC (95% CI)
Training dataset						
Transverse ultrasound model	67.44	83.33	76.29	76.32	76.27	0.825 (0.773–1.00)
Longitudinal ultrasound model	79.07	85.19	82.47	80.95	83.64	0.800 (0.783–0.851)
Fusion model	88.37	87.04	87.63	84.44	90.39	**0.943 (0.867–1.00)**
Validation dataset						
Transverse ultrasound model	50.00	86.36	69.05	76.92	65.52	0.730 (0.651–0.808)
Longitudinal ultrasound model	65.00	90.91	78.57	86.67	74.07	**0.823 (0.800–0.852)**
Fusion model	65.00	90.91	78.57	86.67	74.07	0.800 (0.683–0.917)

CI, confidence interval; PPV, positive predictive value; NPV, negative predictive value; AUC, area under the curve. Bold values indicate the best diagnostic performance in each column.

**Figure 3:**
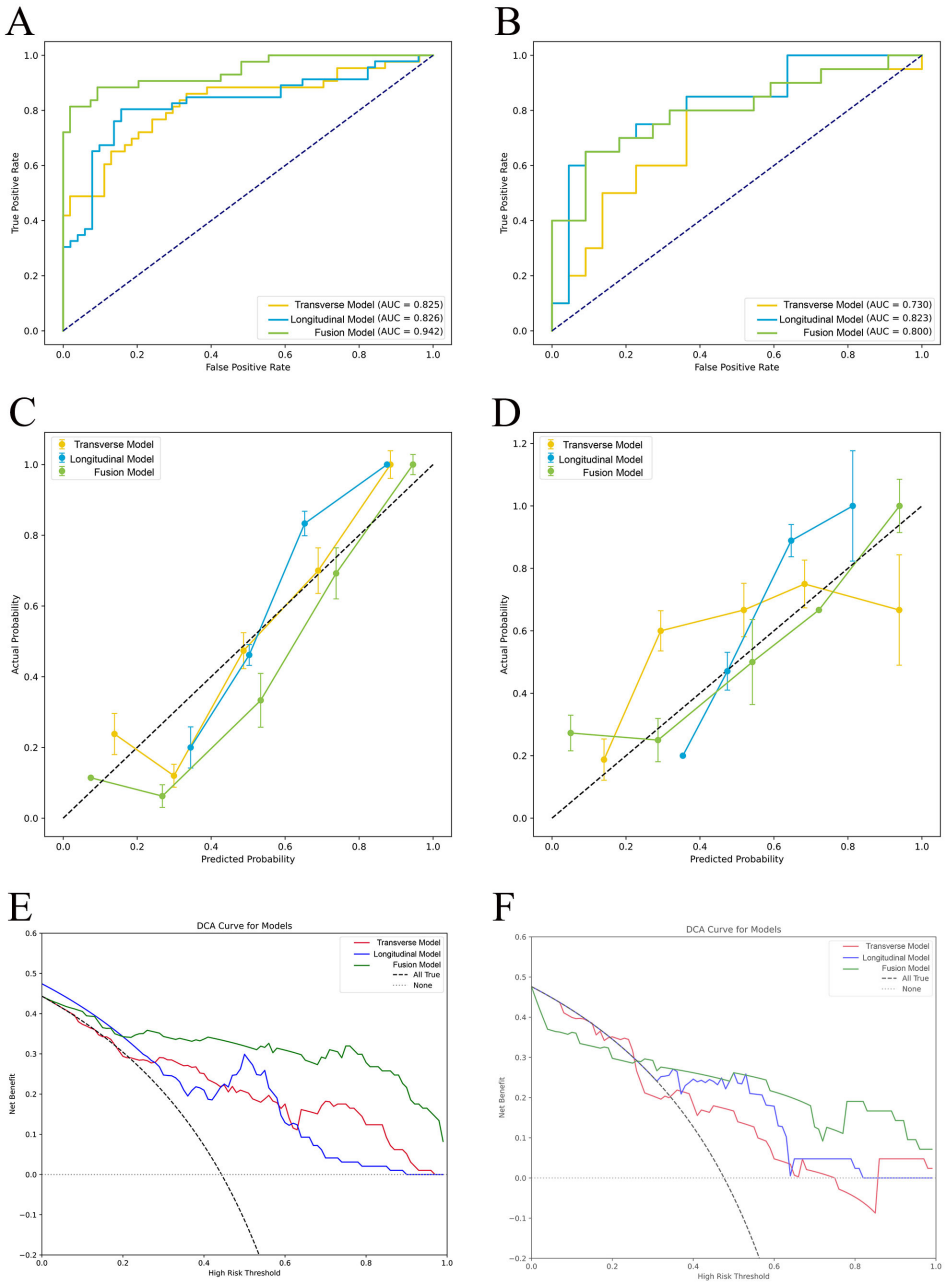
Evaluation of the logistic regression models based on receiver operating characteristic curve analysis **(A, B)**, calibration curves **(C, D)**, and decision curve analysis **(E, F)**, in the training dataset (left) and validation dataset (right).

Calibration curve analysis and DCA were performed for the models in both training and validation datasets. The calibration curves were relatively closer to the ideal line in the training dataset; however, several mild deviations were noted in the validation dataset. In particular, the line for the longitudinal ultrasound model showed downward deviation in the final third of predicted values (**Figures 3C, D**). On DCA, all the three models showed excellent performance in the training dataset. However, the fusion model showed higher net benefit across certain threshold ranges in the validation dataset (**Figures 3E, F**).

### Demonstration of SHAP values

**Supplementary Figure 1** shows the weights of the selected radiomics features. For the transverse model, the predictive performance predominantly relied on the log-sigma features; the wavelet features contributed to a lesser extent. For the longitudinal model, the SHAP diagram showed significant influences of various shapes, grayscale texture, and multi-scale features. The fusion model offered a significant advantage in integrating multi-source information at the feature level. Feature contributions were relatively balanced across the first two models, with no single modality showing predominance.

## Discussion

We herein developed and compared three radiomics models based on transverse, longitudinal, and fusion ultrasound images for differentiating NMBC from mastitis. On the internal hold-out validation set, the longitudinal, transverse, and fusion models achieved AUC values of 0.823, 0.730, and 0.800, respectively, with no significant differences among them (all *P* > 0.05). Notably, across all feature sets, the combined clinical–radiomics model consistently outperformed both the radiomics and clinical variable-based models (AUC: 0.861–0.884), and likelihood ratio tests confirmed that both radiomics features and clinical variables remained significant predictors in the combined model (all *P* ≤ 0.010), indicating complementary and non-redundant contributions to the prediction. These findings suggest that integrating clinical variables with radiomics features provides added diagnostic value beyond either approach alone.

Our findings showed that although the fusion model performed exceptionally well in the training dataset, it could not outperform the transverse or longitudinal models in the validation dataset. This discrepancy is likely attributable to two factors. First, the fusion model may show reduced diagnostic efficacy owing to the limited specificity of the radiomics features derived from the transverse model. Second, the fusion model may be susceptible to overfitting (19, 20), possibly because of the inclusion of a large number of radiomics features in the training dataset.

To further assess the diagnostic relevance of the selected radiomics features, their semantic implications were analyzed based on SHAP value contribution. In the transverse model, log-sigma features predominantly contributed to the prediction. These features were derived from LoG filtering at various scales and highlighted local edge heterogeneity and texture transitions—attributes commonly seen in infiltrative malignant lesions. Wavelet features, which were also prominent in this model, captured multiscale frequency patterns, thus underscoring the complexity of tissue architecture in NMBC. In the longitudinal model, shape features exerted a stronger influence. This suggests that the overall geometry and irregularities of the boundary—albeit subtle—may differ significantly between NMBC and mastitis. Notably, the grayscale texture features, including GLCM and GLSZM, showed differences in pixel intensity distributions, suggesting that malignancies show more complex intralesional heterogeneity than inflammatory lesions. In the fusion model, feature contributions were well-balanced across modalities and feature types; this finding supports the synergistic value of biplanar analysis. Across the three models, texture-related features—particularly those derived from GLSZM and GLCM—consistently ranked among the top contributors, suggesting that intralesional heterogeneity, as captured by grayscale texture patterns, is a key discriminator between NMBC and mastitis regardless of the imaging plane. However, the models differed in terms of their reliance on specific feature types: the transverse model was more dependent on multi-scale edge features (log-sigma and wavelet), whereas the longitudinal model placed greater weight on shape and boundary characteristics; the fusion model, however, integrated both perspectives with balanced contributions. This complementary pattern reinforces the rationale for biplanar analysis, as each plane captures distinct morphological and textural dimensions of the lesions.

Several recent studies have investigated quantitative imaging approaches for breast lesion characterization in contexts relevant to our work. Using multiple machine learning algorithms, Liu et al. (21) developed an intratumoral and peritumoral ultrasound radiomics model for NMBC classification and reported an AUC of 0.870 in their validation cohort. Although our interests are aligned in terms of focus on non-mass lesions, they addressed the broader classification of NMBC rather than the specific challenge of differentiating NMBC from mastitis. In the MRI domain, Zhao et al. (17) used whole-lesion ADC histogram analysis to distinguish between idiopathic granulomatous mastitis and invasive breast carcinoma presenting with non-mass enhancement, and Leithner et al. (16) reported on the utility of DWI-based radiomics signatures for breast cancer characterization. Although these MRI-based studies underscore the potential of quantitative imaging for this differential diagnosis, they are limited by the limited accessibility and high cost of MRI. Regarding ultrasound-based approaches, Wu et al. (22) reported that a combined ultrasound video-based radiomics and clinical model achieved an AUC of 0.926 for differentiating between benign and malignant breast lesions, surpassing the BI-RADS-only model (AUC = 0.737). Similarly, Shi et al. (23) showed that a multimodal ultrasound-based clinical–radiomics nomogram outperformed single-modality approaches for predicting malignancy in solid hypoechoic breast lesions. In addition, contrast-enhanced ultrasound is reportedly valuable in distinguishing granulomatous mastitis from invasive ductal carcinoma (AUC = 0.794 for diffuse enhancement pattern) (24). Notably, our combined clinical–radiomics model achieved AUC values of 0.861–0.884, which are comparable to or exceed the performance reported in these previous studies; this is particularly meaningful considering that our model was aimed at a more challenging and clinically specific task—the differentiation of NMBC from mastitis, where sonographic overlap is particularly pronounced, and to our knowledge, no prior ultrasound radiomics study has specifically targeted this distinction.

A key finding of this study is that radiomics features and clinical variables provide complementary rather than redundant diagnostic information. Our likelihood ratio tests showed that the radiomics score remained a significant predictor after adjusting for age and BI-RADS (*P* = 0.003–0.010), and conversely, clinical variables retained significance after adjusting for radiomics (all *P* < 0.004). This bidirectional independence indicates that neither clinical assessment nor radiomics alone can fully capture the diagnostic information provided by their combination. Notably, the BI-RADS-only model yielded a modest AUC of 0.650 with high specificity (100%) but low sensitivity (30%); this can be attributable to BI-RADS ≥5 being present in only a subset of NMBC cases and the considerable overlap in BI-RADS scores between NMBC and mastitis. The addition of radiomics features substantially improved sensitivity while maintaining reasonable specificity, suggesting that radiomics identifies malignant patterns that are not captured by the conventional BI-RADS framework, which is particularly relevant for non-mass lesions where morphological criteria are less discriminative.

Ultrasound—given its accessibility, cost-effectiveness, and safety—remains a frontline screening and diagnostic modality in breast imaging. However, its diagnostic accuracy, particularly for non-mass breast diseases like NMBC and mastitis, is typically compromised by subjectivity and interoperator variability. This subjectivity is further amplified by the overlap between the sonographic features of NMBC and mastitis, which may lead to misclassification and delayed diagnosis. A potential solution to this issue is the integration of radiomics models into clinical workflows, which introduces objective and reproducible metrics to supplement sonographer assessments. For diseases like NMBC, a radiomics model capable of identifying signatures of malignancy may serve as an early warning tool even in the absence of mass-like features. This added layer of diagnostic support may prompt more targeted follow-up imaging, biopsies, or specialist consultations; this may consequently guide clinical decision-making and improve patient outcomes. Therefore, radiomics may serve as a valuable assistive tool rather than a replacement, potentially enhancing the sensitivity and consistency of ultrasound-based diagnosis of non-mass lesions. Despite its strengths, this study has certain limitations. First, the imaging data were retrospectively analyzed. Although the data were obtained from real-world clinical practice, potential non-systematic bias (e.g., uneven case distribution) may have been introduced during data collection, which may affect the generalizability of the model. Second, the sample size was relatively limited, particularly in the validation cohort; in diverse clinical environments, this may restrict the statistical power and robustness of the model. Third, all ultrasound images were from a single institution and acquired by the same sonography team using the same equipment. Although this helps minimize system variability, it may limit model adaptability when used across different devices and clinical settings. Fourth, despite rigorous quality control and standardized preprocessing, minor differences in image acquisition (e.g., operator-dependent adjustments and subtle variations in device parameters) may have impacted the extraction of high-dimensional features and thus influenced model stability. Fifth, clinical variables were not incorporated in the initial modeling process; however, we have now addressed this by constructing BI-RADS-based, clinical, and combined clinical–radiomics models as supplementary analyses. The significant between-group differences in age and BI-RADS scores reflect the inherent epidemiological characteristics of the diseases; the age-based difference is attributable to the age of peak incidence and was essentially unavoidable. Although most patients with NMBC (48/63, 76.2%) were categorized as having BI-RADS 4 lesions, and mastitis lesions showed significant overlap in BI-RADS scores with those of NMBC, the combined clinical–radiomics model still outperformed the radiomics model, showing the incremental value of incorporating clinical variables. Sixth, the validation set was derived from a random hold-out split of the same institutional cohort rather than from an independent external center. Although this approach allows preliminary evaluation of model generalizability, it does not fully account for cross-institutional variability in imaging protocols, equipment, and patient populations. Prior to clinical implementation, true external validation using independent multicenter datasets is needed.

In the future, several research avenues need to be pursued. First, multicenter external validation with larger cohorts is essential to confirm the generalizability of our findings across diverse imaging protocols and patient populations. Second, adopting automated or semi-automated segmentation methods may enhance reproducibility and facilitate clinical translation. Third, incorporation of deep learning-based feature extraction alongside handcrafted radiomics may capture additional discriminative patterns. Finally, integration of complementary modalities like contrast-enhanced ultrasound or diffusion-weighted imaging into a multimodal radiomics framework may further improve diagnostic performance for this challenging differential diagnosis.

## Conclusion

We found that biplanar ultrasound-based radiomics models have potential for differentiating NMBC from mastitis. Although the fusion model achieved superior diagnostic performance in the training cohort, its advantage was not replicated during internal hold-out validation, indicating limited generalizability. Radiomics holds promise as a valuable adjunct to conventional ultrasound interpretation and may represent a more objective approach for the early identification of non-mass breast malignancies. The integration of multimodal features, such as imaging characteristics and clinical parameters, can further enhance the comprehensive diagnostic performance of predictive models, as demonstrated by the superior performance of the combined clinical–radiomics model in our supplementary analysis.

## Data Availability

The raw data supporting the conclusions of this article will be made available by the authors, without undue reservation.

## Supplementary material

Supplementary Figure 1 SHAP value plots showing the relative importance and direction of influence of key features on the output of three distinct models. Each plot lists features (such as imaging radiomics features and shape descriptors) and their corresponding SHAP values along the y and x axes, respectively; positive and negative SHAP values indicate facilitatory and inhibitory effects on the model output, respectively.

Supplementary Figure 2 Evaluation of the clinical variable-based, radiomics-based, and clinical-radiomics models based on receiver operating characteristic curve analysis **(A–C)**, calibration curves **(D–F)**, and decision curve analysis **(G–I)**, in the longitudinal (left), transverse (middle), and fusion (right) feature sets.
