# Supplementary figures and images for: Radiomics analysis of biplanar ultrasound images can discriminate non-mass breast carcinoma from mastitis
Source: Front Oncol. 2026 Jul 1;16:1785714. doi: 10.3389/fonc.2026.1785714 (PMC13368669; doi:10.3389/fonc.2026.1785714)

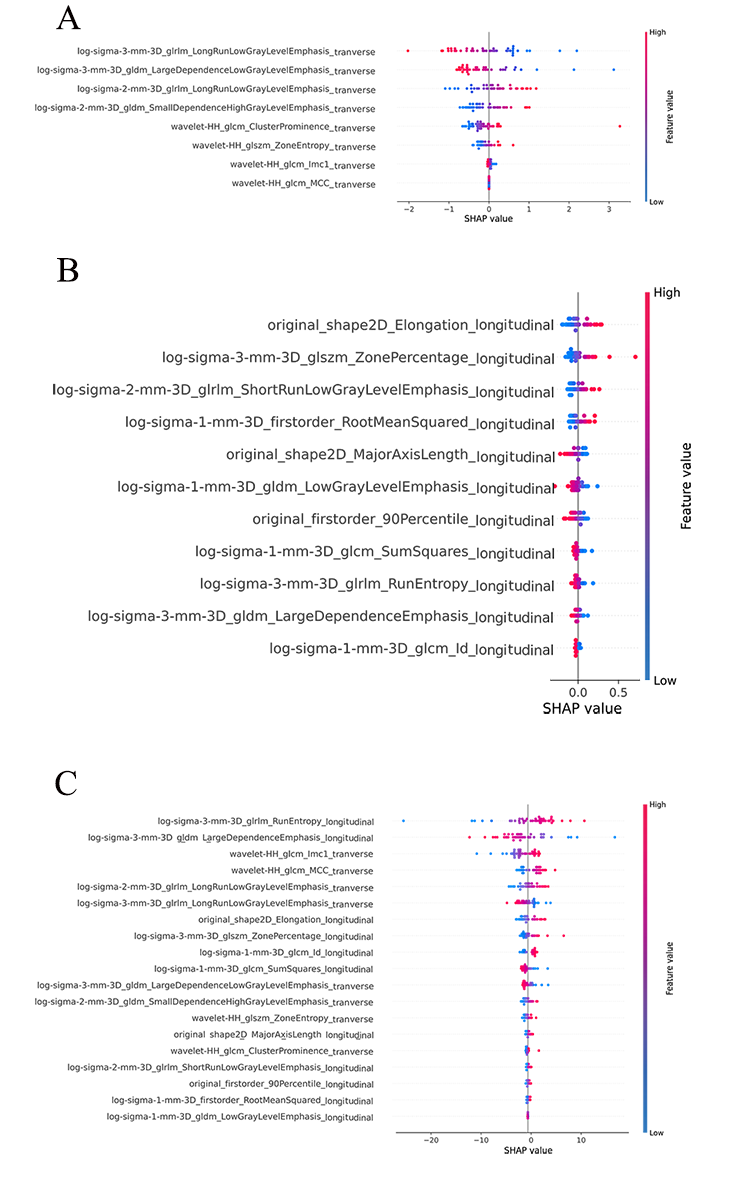

Supplement: Supplementary Figure 1 — SHAP value plots showing the relative importance and direction of influence of key features on the output of three distinct models. Each plot lists features (such as imaging radiomics features and shape descriptors) and their corresponding SHAP values along the y and x axes, respectively; positive and negative SHAP values indicate facilitatory and inhibitory effects on the model output, respectively. [file Image1.tif]

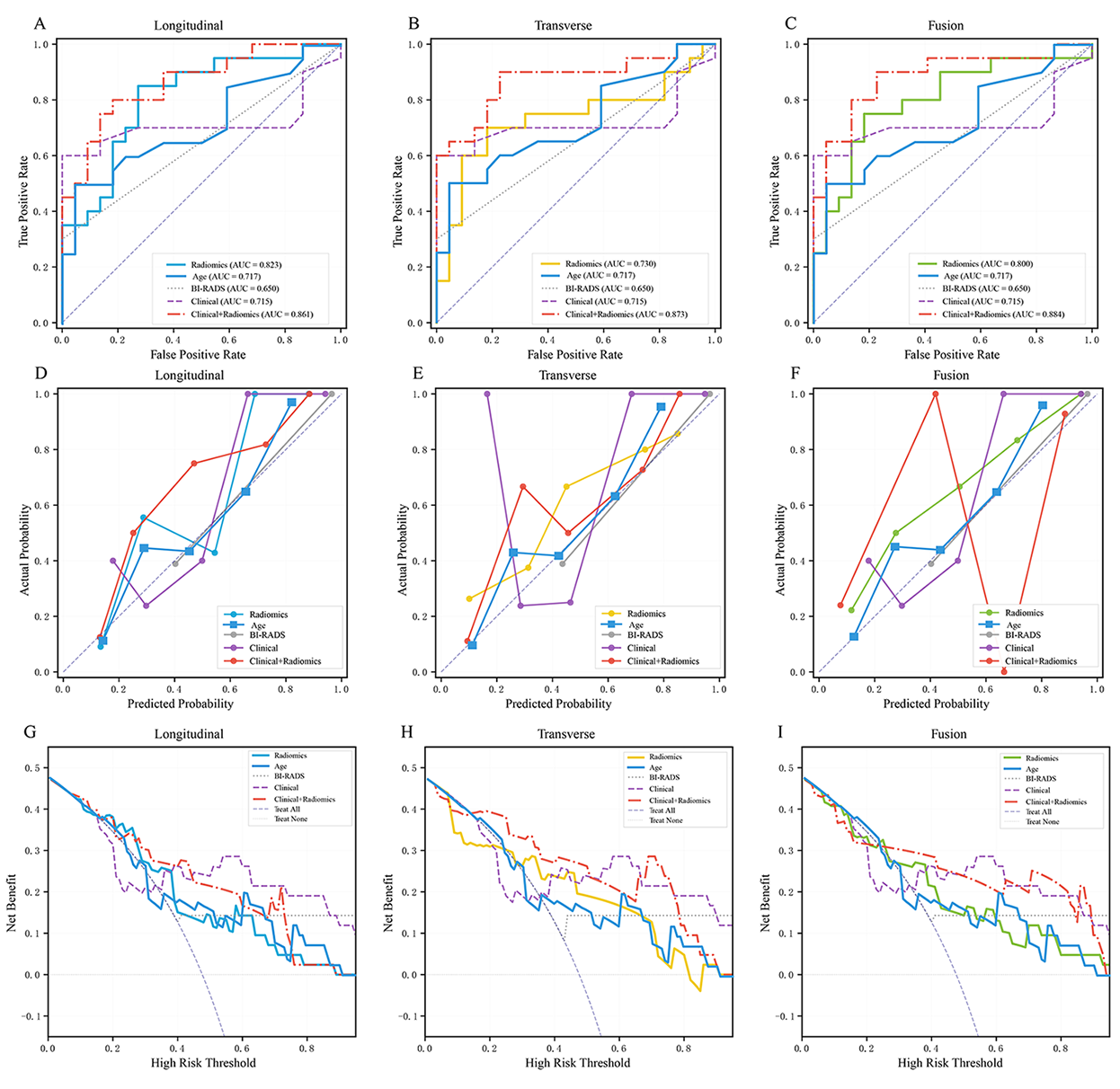

Supplement: Supplementary Figure 2 — Evaluation of the clinical variable-based, radiomics-based, and clinical-radiomics models based on receiver operating characteristic curve analysis (A–C), calibration curves (D–F), and decision curve analysis (G–I), in the longitudinal (left), transverse (middle), and fusion (right) feature sets. [file Image2.tif]
