# Supplementary material for: Radiomics analysis of biplanar ultrasound images can discriminate non-mass breast carcinoma from mastitis
Source: Front Oncol. 2026 Jul 1;16:1785714. doi: 10.3389/fonc.2026.1785714 (PMC13368669; doi:10.3389/fonc.2026.1785714)
Supplement: Supplementary file 3 [file Table1.docx]

**Supplementary Table S1.** Diagnostic performance of clinical variable-based, radiomics-based, and clinical-radiomics models in the validation dataset (n = 42)

| **Model** | **Sensitivity (%)** | **Specificity (%)** | **Accuracy (%)** | **PPV (%)** | **NPV (%)** | **AUC (95% CI)** |
| --- | --- | --- | --- | --- | --- | --- |
| **Clinical variable-based models** |  |  |  |  |  |  |
| age | 60.00 | 77.27 | 69.05 | 70.59 | 68.00 | **0.717 (0.553–0.861)** |
| BI-RADS | 30.00 | 100.00 | 66.67 | 100.00 | 61.11 | 0.650 (0.550–0.750) |
| Clinical (age + BI-RADS) | 60.00 | 90.91 | 76.19 | 85.71 | 71.43 | 0.715 (0.524–0.886) |
| **Radiomics-based models** |  |  |  |  |  |  |
| Transverse Radiomics | 50.00 | 86.36 | 69.05 | 76.92 | 65.52 | 0.730 (0.651–0.808) |
| Longitudinal Radiomics | 65.00 | 90.91 | 78.57 | 86.67 | 74.07 | **0.823 (0.800–0.852)** |
| Fusion Radiomics | 65.00 | 90.91 | 78.57 | 86.67 | 74.07 | 0.800 (0.683–0.917) |
| **Clinical-Radiomics models** |  |  |  |  |  |  |
| Transverse Clinical + Radiomics | 70.00 | 86.36 | 78.57 | 82.35 | 76.00 | 0.873 (0.733–0.970) |
| Longitudinal Clinical + Radiomics | 55.00 | 90.91 | 73.81 | 84.62 | 68.97 | 0.861 (0.730–0.957) |
| Fusion Clinical + Radiomics | 65.00 | 86.36 | 76.19 | 81.25 | 73.08 | **0.884 (0.757–0.975)** |

**Note:** CI, confidence interval; PPV, positive predictive value; NPV, negative predictive value; AUC, area under the curve. BI-RADS categories were binarized as 4A/4B/4C = 0 and 5/6 = 1. Clinical model incorporated age and binary BI-RADS category. All models were developed using logistic regression. 95% CIs for AUC were derived from 2,000 bootstrap resamples.

**Table S2 Radiomics features of the models for NMBC and mastitis**

| ***Transverse Model*** |
| --- |
| log-sigma-3-mm-3D_glrlm_LongRunLowGrayLevelEmphasis_transverse |
| log-sigma-3-mm-3D_gldm_LargeDependenceLowGrayLevelEmphasis_transverse |
| log-sigma-2-mm-3D_glrlm_LongRunLowGrayLevelEmphasis_transverse |
| log-sigma-2-mm-3D_gldm_SmallDependenceHighGrayLevelEmphasis_transverse |
| wavelet-HH_glcm_ClusterProminence_transverse |
| wavelet-HH_glszm_ZoneEntropy_transverse |
| wavelet-HH_glcm_lmc1_transverse |
| wavelet-HH_glcm_MCC_transverse |
| ***Longitudinal Model*** |
| original_shape2D_Elongation_longitudinal |
| log-sigma-3-mm-3D_glszm_ZonePercentage_longitudinal |
| log-sigma-2-mm-3D_glrlm_ShortRunLowGrayLevelEmphasis_longitudinal |
| log-sigma-1-mm-3D_firstorder_RootMeanSquared_longitudinal |
| original_shape2D_MajorAxisLength_longitudinal |
| log-sigma-1-mm-3D_gldm_LowGrayLevelEmphasis_longitudinal |
| original_firstorder_90Percentile_longitudinal |
| log-sigma-1-mm-3D_glcm_SumSquares_longitudinal |
| log-sigma-3-mm-3D_glrlm_RunEntropy_longitudinal |
| log-sigma-3-mm-3D_gldm_LargeDependenceEmphasis_longitudinal |
| log-sigma-1-mm-3D_glcm_Id_longitudinal |
| ***Fusion Model*** |
| Rad feature from Model 1 & 2 |

NMBC, Non-mass breast carcinoma; _transverse, radiomics features from transverse ultrasound images; _longitudinal, radiomics features from longitudinal ultrasound images
